# A Fast Response Highly Selective Probe for the Detection of Glutathione in Human Blood Plasma

**DOI:** 10.3390/s120505940

**Published:** 2012-05-08

**Authors:** Yixing Guo, Xiaofeng Yang, Lovemore Hakuna, Aabha Barve, Jorge O. Escobedo, Mark Lowry, Robert M. Strongin

**Affiliations:** 1 Department of Chemistry, Portland State University, Portland, OR 97201, USA; E-Mails: yixing@pdx.edu (Y.G.); lhakun@pdx.edu (L.H.); aabha@pdx.edu (A.B.); jescobed@pdx.edu (J.O.E.); mlowry.chem@gmail.com (M.L.); 2 Key Laboratory of Synthetic and Natural Functional Molecule Chemistry of Ministry of Education, Institute of Analytical Sciences, College of Chemistry & Materials Science, Northwest University, Xi'an 710069, China; E-Mail: xfyang@nwu.edu.cn

**Keywords:** fluorescent probe, glutathione, micelle-catalyzed, selectivity

## Abstract

A fluorescent probe for glutathione (GSH) detection was developed. Our study indicates a possible mechanism which couples a conjugate addition and micelle-catalyzed large membered ring formation/elimination sequence. This method enables excellent selectivity towards GSH over other biological thiols such as cysteine (Cys) and homocysteine (Hcy). The proposed method is precise with a relative standard deviation (R.S.D) lower than 6% (*n* = 3) and has been successfully applied to determine GSH in human plasma with recoveries between 99.2% and 102.3%.

## 1. Introduction

Glutathione (GSH) serves as an antioxidant and an important indicator of cellular oxidative stress [1]. Aberrant levels of GSH have been associated with a number of diseases, including cancer, AIDS, Alzheimer's and cardiovascular disease [2]. Fluorescent and colorimetric probes for the detection of thiols have been widely reported [3]. However, indicators that are selective for GSH and not generally selective for sulfhydril-containing compounds are relatively rare. Although several recent papers claim GSH selectivity, the indicators display significant responses to Cys and other related nucleophilic thiols [4–7]. A promising GSH-selective probe for selective intracellular imaging applications has been developed by Shao *et al.* [8]. In addition, innovative nanoparticle or polymeric indicators for GSH also exhibit high selectivity, but to date they have not been successfully used in biological media, as they either are based on toxic CdSe or require the handling of highly toxic mercury salts to function [9,10].

**Scheme 1.**
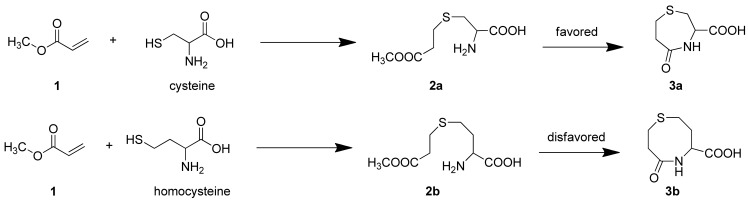
The condensation reaction of acrylates with Cys and Hcy to form 3-carboxy-5-oxoperhydro-1,4-thiazepines (**3**).

It has been reported that Cys reacts with acrylates to generate thioethers that undergo an intramolecular cyclization reaction to yield, for example, 3-carboxy-5-oxoperhydro-1,4-thiazepine (**3a**) [11]. In the case of Hcy, **2b** should be easily obtained [12], however, the intramolecular cyclization reaction to form a eight-membered ring (**3b**) is kinetically disfavored, compared with the formation of seven membered ring (**3a**) which would result from Cys (Scheme 1) [13–15].

Recently, a new design for a fluorescent probe capable of distinguishing Cys and Hcy was developed in our lab. A (hydroxymethoxyphenyl)benzothiazole (HMBT)-based probe functioned based on a combined photo-induced electron transfer (PET) and excited-state intramolecular proton transfer (ESIPT) mechanism [16]. More recently, we developed a seminaphthofluorescein (SNF)-based probe for the long wavelength, highly selective detection of Cys. It couples a conjugate addition/cyclization mechanism to a xanthene dye spirolactone-opening reaction [17].

However, to achieve the highly selective detection of GSH over Cys and Hcy, one would need to overcome the formidable challenge of favoring the formation of a larger (12-membered ring in the case of GSH) over a 7- and 8-membered ring (as in **3a** and **3b**). Interestingly, it had been reported earlier that cetyltrimethylammonium bromide (CTAB) micelles can catalyze the intramolecular ring closure of larger rings, reversing the expected kinetics based on ring size [18]. It is also known that GSH binds the surface of cationic CTAB micelle [19]. We reasoned that a relatively smaller and more planar dye as compared to the HMBT and SNF probes used previously may interact more with the micelle and thus promote highly selective detection of GSH [20]. We thus herein report the successful use of a resorufin-based probe for the highly selective detection of GSH in CTAB medium.

## 2. Experimental Section

### 2.1. Materials and Instruments

All chemicals were purchased from Sigma-Aldrich or Acros and used without further purification.^1^H-NMR and ^13^C-NMR spectra were recorded on a Bruker AMX-400 NMR spectrometer, using TMS as an internal standard. ESI-HRMS (high resolution mass spectrometry) spectra were obtained on a Thermo Electron LTQ Orbitrap hybrid mass spectrometer. UV-visible spectra were collected on a Cary 50 UV-Vis spectrophotometer; Fluorescence spectra were collected on a Cary Eclipse (Varian, Inc.) fluorescence spectrophotometer with slit widths set at 5 nm for both excitation and emission, respectively. The high voltage of the fluorescence spectrophotometer was set at 500 V for the resorufin-based probe **4**. The pH measurements were carried out with an Orion 410A pH meter.

### 2.2. Synthesis of Probe **4**

**Scheme 2.**
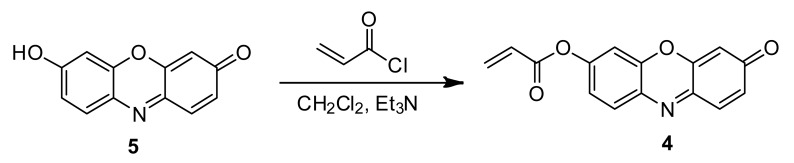
Synthesis of **4**.

Probe **4** was synthesized in a one-step reaction of resorufin with acryloyl chloride (Scheme 2). To a solution of resorufin (220 mg) and Et_3_N (1.5 equiv) in 15 mL of anhydrous CH_2_Cl_2,_ acryloyl chloride (2.0 equiv in 5 mL of CH_2_Cl_2_) is added dropwise at 0 °C. After stirring at this temperature for 60 min, the resulting mixture is allowed to cool to room temperature and stirred overnight. The mixture is diluted with CH_2_Cl_2_ (20 mL), washed with H_2_O (10 mL × 3) and dried over anhydrous Na_2_SO_4_. The solvent is removed by evaporation to afford an orange solid (172 mg, 70% yield). ^1^H NMR (CDCl_3_, 400 MHz), δ (ppm): 7.92 (d, 1H, *J* = 8.7 Hz), 7.59 (d, 1H, *J* = 9.8 Hz), 7.49 (s, 1H), 7.33 (dd, 1H, *J*_1_ = 2.4 Hz, *J*_2_ = 2.5 Hz), 6.83 (dd, 1H, *J*_1_ = 2.0 Hz, *J*_2_ = 2.0 Hz), 6.63 (d, 1H, *J* = 17.2 Hz), 6.49 (m, 1H), 6.24 (s, 1H), 6.22 (d, 1H, *J* = 1.0 Hz). ^13^C NMR (CDCl_3_, 100 MHz), δ 186.48, 163.84, 153.24, 148.87, 147.78, 143.55, 134.85, 134.26, 134.15, 131.22, 131.16, 127.18, 118.82, 109.94, 105.86. ESI-MS *m/z* = 268.0833 [M+H]^+^, calc. 268.0810 for C_15_H_10_NO_4_.

## 3. Results and Discussion

### 3.1. Intrinsic Cysteine Selectivity in the Absence of Surfactant

**Figure 1.**
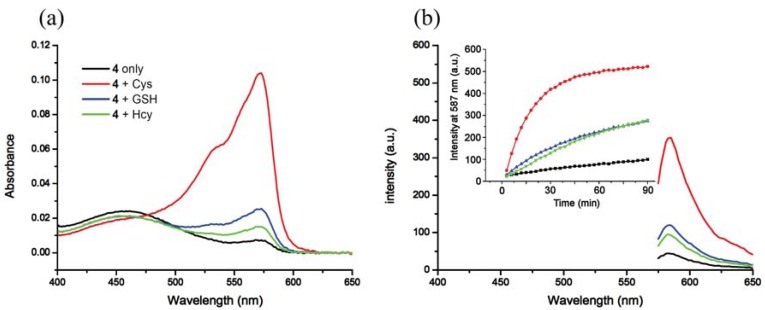
Spectral response of **4** towards various thiols. (**a**) Absorption spectra of **4** (2.5 μM) upon addition of thiols (2 equiv) in phosphate buffer (pH 7.4, 50 mM) at 20 min; (**b**) Fluorescence spectra of **4** (2.5 μM) upon addition of thiols (2 equiv) in phosphate buffer (pH 7.4, 50 mM) (λ_ex_ = 565 nm) at 20 min. The inset shows time-dependent fluorescence changes (λ_em_ = 587 nm). The legends in (**a**) correspond to (**b**) as well.

**Scheme 3.**
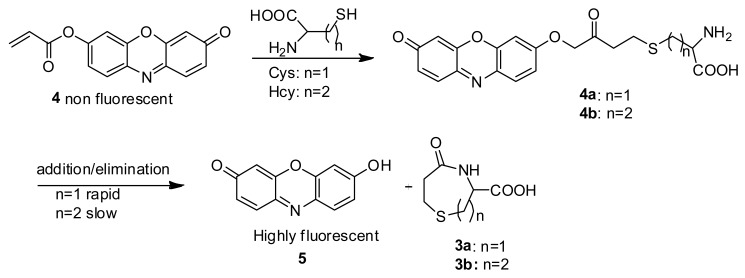
Proposed mechanism for the intrinsic response of **4** towards Cys.

In buffer without a surfactant, as expected, **4** shows excellent selectivity towards Cys due to the reported kinetically favored 7-membered ring formation. Upon mixing Cys with **4**, the conjugate addition product **4a** is formed, which undergoes a rapid cyclization reaction to produce **3a** while releasing the free resorufin (Scheme 3). A significant color change and fluorescence enhancement can be observed in response to Cys (Figure 1). In the case of Hcy, because it has an additional methylene group in its side chain, a kinetically less favored 8-membered ring would form. As for GSH, 1,4-addition of thiols to the α,β-unsaturated carbonyl moieties of **4** can occur readily; however, the ensuing intramolecular cyclization similar to that of Cys cannot proceed without the presence of surfactant. No significant color formation or fluorescence response is seen in the case of Hcy or GSH under these conditions.

The time course of the fluorescence assay is shown in Figure 1(b). It can be seen that the fluorescence upon reaction with Cys increases with time and reaches a plateau after about 90 min, whereas for Hcy and GSH the reactions are significantly slower. The sensitivity and linearity of the response of **4** towards Cys was also investigated. Linear response with submicromolar sensitivity was observed (See Figure S5 in the Electronic Supplementary Information). The reaction products between **4** and thiols were further studied using HRMS (See Figures S6 and S7 in the Electronic Supplementary Information).

### 3.2. Surfactant Mediated Modification of the Intrinsic Selectivity

**Figure 2.**
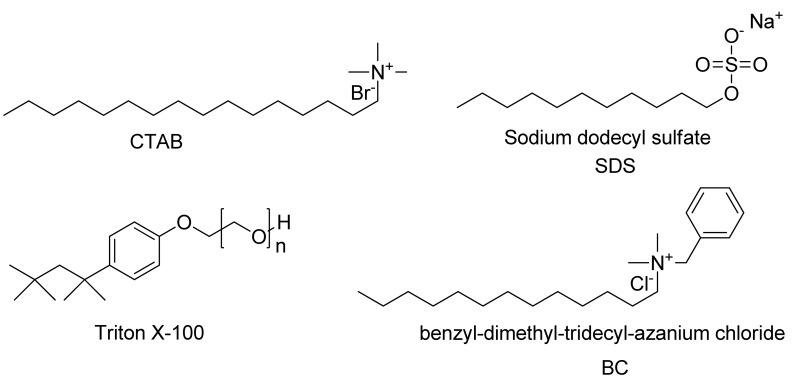
Structures of surfactants employed.

**Figure 3.**
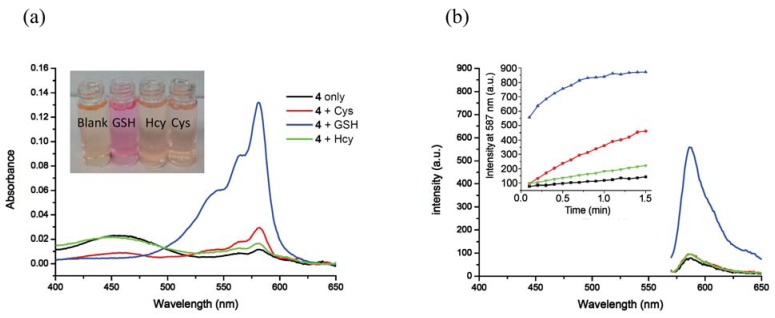
Surfactant-mediated responses of solutions containing **4-**CTAB towards various thiols at pH 7.4. (**a**) Absorption spectra of **4** (2.5 μM) upon addition of thiols (2 equiv) in 2.0 mM CTAB media buffered at pH 7.4 (phosphate buffer, 50 mM). The inset shows color changes of the solution of **4** (10 μM) upon addition of thiols (2 equiv) in 2.0 mM CTAB media buffered at pH 7.4 (phosphate buffer, 50 mM). The picture was taken immediately upon addition of CTAB; (**b**) Fluorescence spectra (λ_ex_ = 565 nm) of **4** (2.5 μM) upon addition of thiols (2 equiv) in 2.0 mM CTAB media buffered at pH 7.4 (phosphate buffer, 50 mM). Spectra were taken immediately upon addition of CTAB. The inset shows time-dependent fluorescence changes (λ_em_ = 587 nm) of the same system. The legends in (**a**) correspond to (**b**) as well.

Inclusion of surfactants (see Figure 2) dramatically alters the intrinsic selectivity of **4**. For example, solutions of GSH, Cys and Hcy (2 equiv) with the resorufin-acrylate probe **4** in pH 7.4 phosphate buffer generate the characteristic strong pink color immediately upon addition of CTAB only in the presence of GSH (Figure 3(a)). Other amino acids, such as Hcy and Cys, did not exhibit significant changes under the same conditions. This interesting feature indicates that **4**-CTAB system can serve as a selective visual inspection dosimeter for GSH. Corresponding fluorescence increases were also observed (Figure 3(b)). Fluorescence upon reaction with GSH reaches a plateau in less than 2 min.

**Figure 4.**
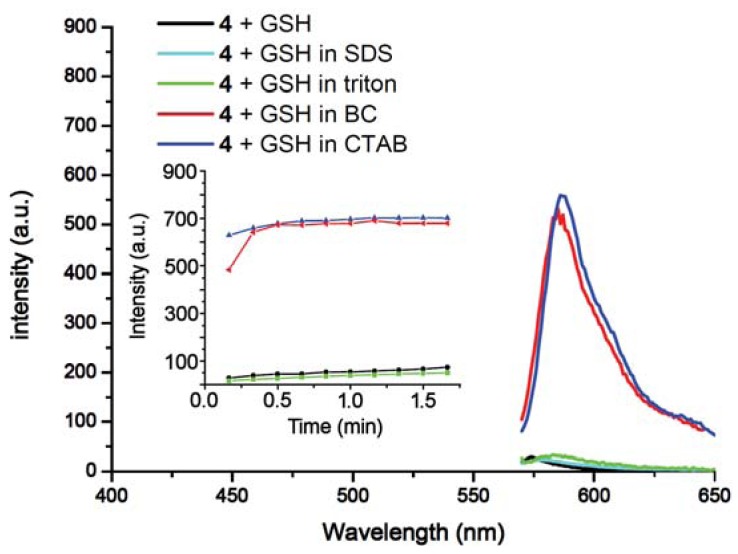
Surfactant-mediated responses of **4** towards GSH. Fluorescence spectra (λ_ex_ = 565 nm) of **4** (2.5 μM) upon addition of GSH (2 equiv) in pH 7.4 buffered (phosphate buffer, 50 mM) surfactant media [(SDS, 10.0 mM), (Triton X-100, 0.3 mM), (BC, 0.05 mM) and (CTAB, 2.0 mM)]. Spectra were taken immediately upon addition of surfactants. The inset shows time-dependent fluorescence changes (λ_em_ = 587 nm) of the same system.

To study the effects of different surfactants, SDS, BC and Triton X-100 were evaluated. It was found that **4** serves as an outstanding indicator for GSH only in the presence of cationic surfactants such as BC and CTAB (Figure 4). Negatively charged SDS suppressed the response of **4** towards all three thiols; while the non-ionic surfactant Triton X-100 enhanced the selectivity towards Cys resulting in zero response for Hcy or GSH (See Figure S8 in the Electronic Supplementary Information). The result indicates that selectivity of **4** is tunable by simply changing the reaction media rather than the chromogen.

**Figure 5.**
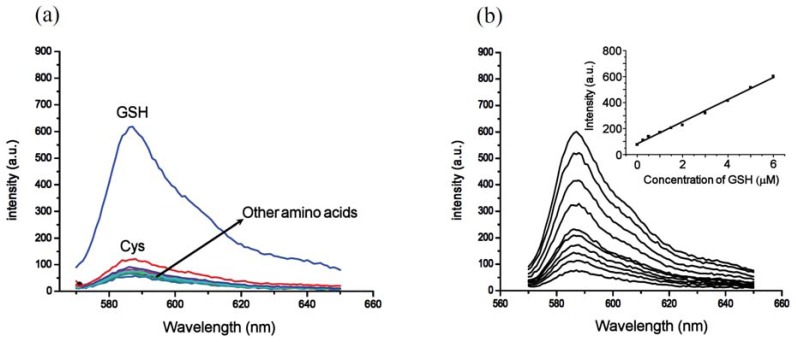
Surfactant mediated response of the **4-**CTAB system toward 21 thiols and amino acids. (**a**) Fluorescence spectra (λ_ex_ = 565 nm) of **4** (2.5 μM) upon addition of Cys, Hcy, GSH, leucine, proline, arginine, histidine, valine, methionine, threonine, glutamine, alanine, aspartic acid, norleucine, isoleucine, lysine, tryptophan, tyrosine, phenylalanine, cystine and homocystine (2 equiv) in 2.0 mM CTAB media at pH 7.4 (phosphate buffer, 50 mM). Spectra were taken immediately upon the addition of thiols and other amino acids; (**b**) Fluorescence spectra (λ_ex_ = 565 nm) of **4** (2.5 μM) upon addition of increasing concentrations of GSH in 2.0 mM CTAB media at pH 7.4 (phosphate buffer, 50 mM). Spectra were taken immediately upon addition of CTAB. The inset shows a linear relationship between fluorescence intensity (λ_ex_ = 565 nm, λ_em_ = 587 nm) and GSH concentration (0–6 μM) with a correlation coefficient of 0.998.

To further evaluate the selectivity of the **4-**CTAB system towards GSH, control experiments using a series of other amino acids were performed measuring the fluorescence responses. Other amino acids produced no significant fluorescence enhancement as compared to GSH, further demonstrating the excellent selectivity for GSH (Figure 5(a)). Moreover, significant fluorescence enhancement can still be observed for GSH even in the presence of excess Cys (see Figure S9 in the Electronic Supplementary Information). Additionally, the fluorescence enhancement of the 4-CTAB system displayed submicromolar sensitivity and a linear relationship with the concentration of GSH over a wide range (Figure 5(b)). The system functions well within the range of typical GSH concentrations in human plasma [21].

**Scheme 4.**
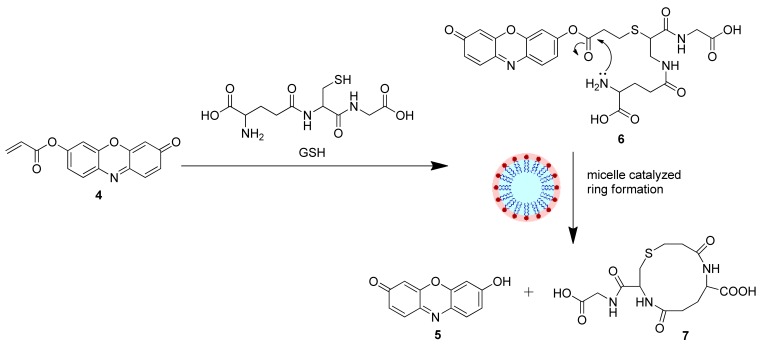
Proposed mechanism for the surfactant mediated response of **4** towards GSH.

Based on the above results, we propose a plausible mechanism for the GSH selectivity of the 4-CTAB system that involves the conjugate addition of GSH to **4** to generate **6**, which in turn undergoes an intramolecular cyclization/elimination reaction sequence catalyzed by the CTAB micelle releasing the free resorufin dye (Scheme 4), resulting in the recovery of the fluorescence. Formation of the 12-membered ring (**7**) and the free resorufin dye were further investigated via HRMS (See Figures S10, S11 in the Electronic Supplementary Information).

### 3.3. Determination of GSH in Human Plasma Using the 4-CTAB System

**Figure 6.**
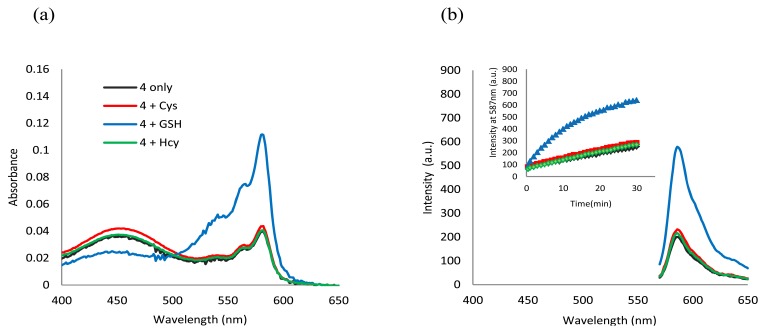
Surfactant mediated response of the **4-**CTAB system towards various thiols at pH 6.0. (**a**) Absorption spectra of **4** (2.5 μM) upon addition of thiols (2 equiv) in 2.0 mM CTAB media buffered at pH 6 (phosphate buffer, 50 mM) at 20 min; (**b**) Fluorescence spectra (λ_ex_ = 565 nm) of **4** (2.5 μM) upon addition of thiols (2 equiv) in 2.0 mM CTAB media buffered at pH 6 (phosphate buffer, 50 mM) at 20 min. The inset shows time-dependent fluorescence changes (λ_em_ = 587 nm) of the same system. The legends in (**a**) correspond to (**b**) as well.

While the **4**-CTAB-GSH signal is high at pH 7.4 (see above), it is known that acrylate esters are susceptible to hydrolysis at high pH and that GSH is also relatively unstable under the same conditions (half-life of 9 hours at pH 7.4 compared to 16 hours at pH 6.5) [22]. In this regard, spectral behavior of the **4**-CTAB system was investigated over a wide range of pH values (pH 5.5 to pH 8) and its response to the various thiols was studied at lower pH values. As expected, the acrylate-based probe **4** showed the greatest background signal at the higher pH values investigated. The **4**-CTAB system was most stable at lower pH values (See Figure S12 in the Electronic Supplementary Information). The reaction with thiols at pH 6 is slightly less rapid as compared to pH 7.4; however, the selectivity towards GSH was further enhanced through the complete elimination of any residual Cys and Hcy response (Figure 6). Thus pH 6 was chosen for further application studies in plasma.

**Figure 7.**
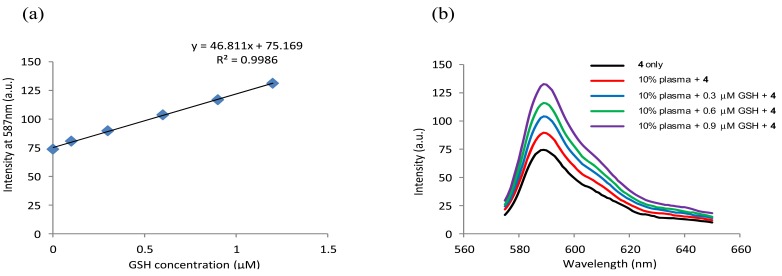
Determination of GSH in human plasma using the **4-**CTAB system. (**a**) Calibration curve of **4** (1.0 μM) in 2.0 mM CTAB media buffered at pH 6 (phosphate buffer, 50 mM) showing a linear relationship between fluorescence intensity (λ_ex_ = 565 nm, λ_em_ = 587 nm) and GSH concentration (0.1–1.2 μM); (**b**) Fluorescence spectra (λ_ex_ = 565 nm) of **4** (1.0 μM) upon addition of GSH (0–1.0 μM) to 10% deproteinized plasma diluted with 2.0 mM CTAB media buffered at pH 6.0 (phosphate buffer, 50 mM). The calibration curve and emission spectra were collected 8 min after mixing.

The detection of GSH was performed in 10% deproteinized human plasma. Plasma proteins were precipitated using MeCN (two thirds of the reconstitution volume) and removed by centrifugation at 4,000 rpm for 30 min. The supernatant liquid was diluted [23] and added to a solution of **4** (1 μM) in phosphate buffer (pH 6, 50 mM) in the presence of 2.0 mM CTAB. The fluorescence response of replicate (*n* = 3) samples and GSH spiked samples were monitored as above and the GSH content in the plasma sample was determined from the regression equation of a standard calibration curve (Figure 7). The GSH content of the plasma sample was determined to be 3.24 ± 0.14 μM, which is well within the reported GSH concentration range for human plasma samples from healthy individuals [21]. Recoveries of the known spiked amounts of GSH were between 99.2% and 102.3% with a generally satisfactory precision (Table 1). These results revealed the potential applicability and reliability of using **4** in quantitative detection of GSH in human plasma. In addition, Cys exhibited significant interference with the GSH determination (See Figure S13 in the Electronic Supplementary Information).

## 4. Conclusions

Probe **4** can function as a simple new indicator for the highly selective and sensitive detection of GSH. This embodies a sensing mechanism that couples a conjugate addition reaction and a micelle-promoted 12-membered ring formation/elimination sequence. Due to the lack of fluorescence probes for the highly selective detection of GSH over other thiols in blood plasma without the use of biohazardous materials, the discovery of **4** is highly significant. A new class of fluorescent probes for GSH may be developed through this new mechanism. Besides, the method can be tuned to afford Cys selectivity if needed as well.

## Supplementary Material



## Figures and Tables

**Table 1.** Determination of GSH content in 10% deproteinized human plasma samples.

**Sample**	**GSH Spiked (μM)**	**GSH Measured (μM)**	**Recovery (%)**	**RSD (%)**
10% Plasma	0	0.324	-	4.42
10% Plasma + GSH	0.3	0.631	102.3	4.90
10% Plasma + GSH	0.6	0.919	99.2	4.24
10% Plasma + GSH	0.9	1.224	100.0	5.88
