# Supplementary material for: A Fast Response Highly Selective Probe for the Detection of Glutathione in Human Blood Plasma
Source: Sensors (Basel). 2012 May 8;12(5):5940–50. doi: 10.3390/s120505940 (PMC3386722; doi:10.3390/s120505940)
Supplement: Supplementary file 1 [file sensors-12-05940-s001.pdf]

OPEN ACCESS
sensors
ISSN 1424-8220
www.mdpi.com/journal/sensors

Supplementary Information

A Fast Response Highly Selective Probe for the Detection of Glutathione in Human Blood Plasma. *Sensors* 2012, *12*, 5940-5950

Yixing Guo [1], Xiaofeng Yang [2], Lovemore Hakuna [1], Aabha Barve [1], Jorge O. Escobedo [1], Mark Lowry [1] and Robert M. Strongin [1,*]

1 Department of Chemistry, Portland State University, Portland, OR 97201, USA; E-Mails: yixing@pdx.edu (Y.G.); lhakun@pdx.edu (L.H.); aabha@pdx.edu (A.B.); jescobed@pdx.edu (J.O.E.); mlowry.chem@gmail.com (M.L.)

2 Key Laboratory of Synthetic and Natural Functional Molecule Chemistry of Ministry of Education, Institute of Analytical Sciences, College of Chemistry & Materials Science, Northwest University, Xi'an 710069, China; E-Mail: xfyang@nwu.edu.cn

* Author to whom correspondence should be addressed; E-Mail: strongin@pdx.edu; Tel.: +1-503-725-9724.

Figure S1. ^{1}H NMR (400 MHz) spectrum of **4** in DMSO-d_6.

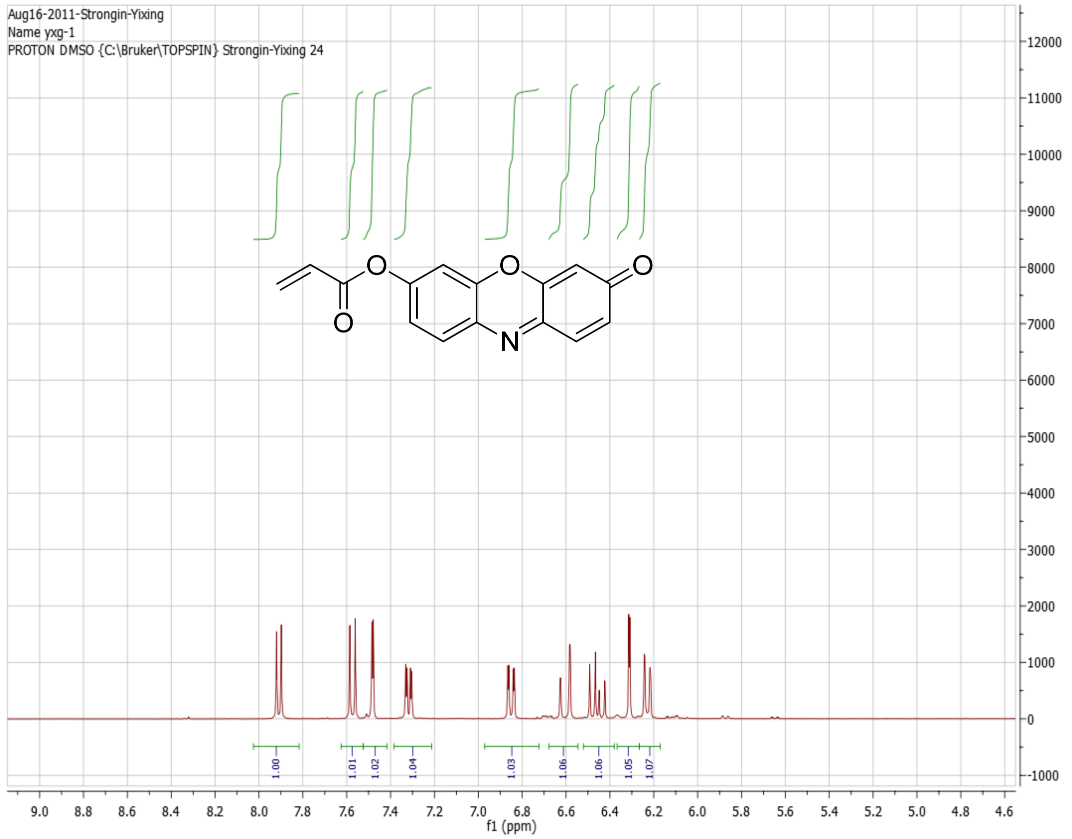

Figure S2. ^{13}C NMR (100 MHz) spectrum of **4** in DMSO-d_6.

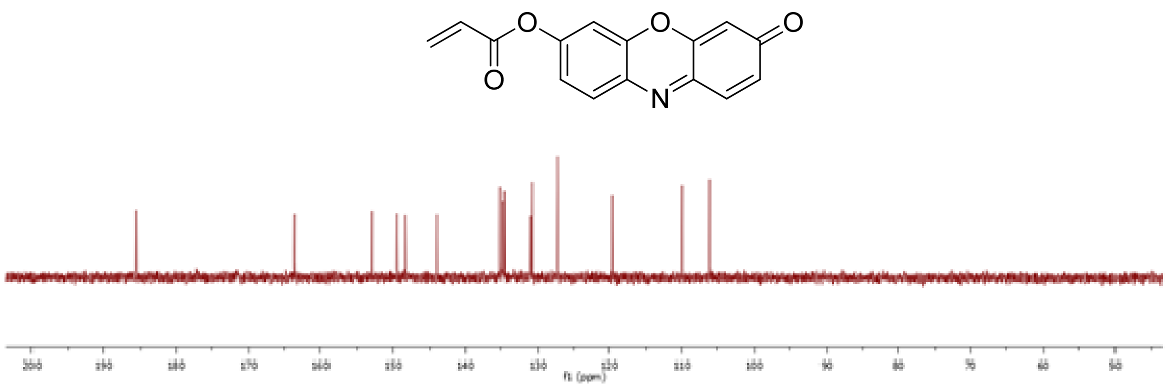

Figure S3. HRMS of **4**.

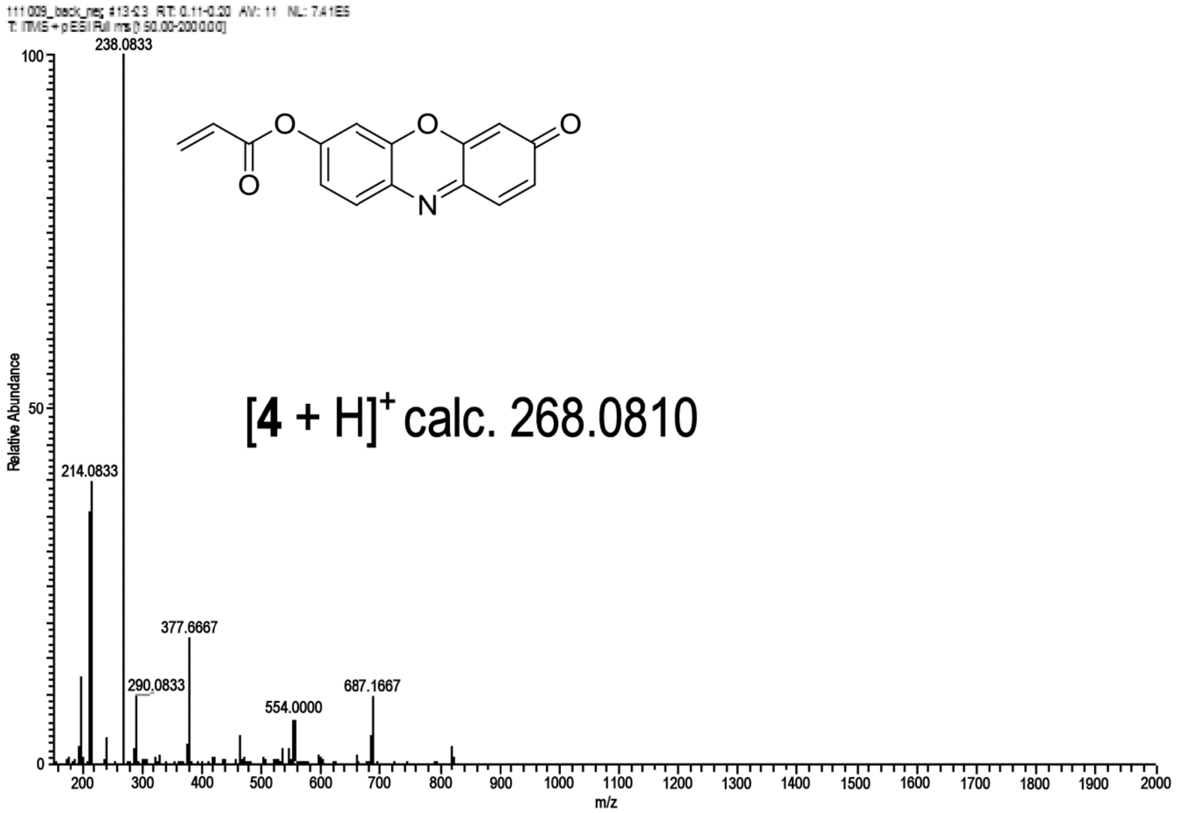

Figure S4. Absorption spectrum of **4** (2.5 μM) in 50 mM phosphate buffer (pH 7.4).

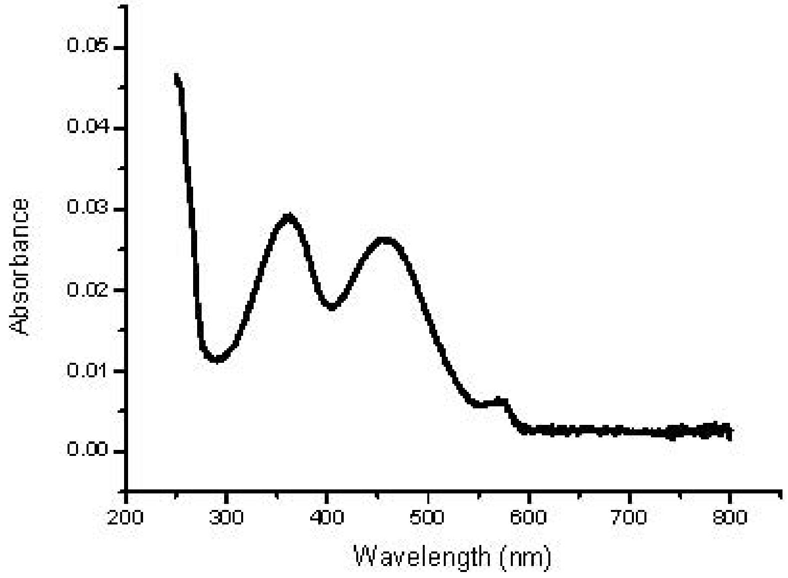

Figure S5. Intrinsic linear response of **4** towards Cys. Fluorescence spectra (λ_{ex} = 565 nm) of **4** (2.5 μM) upon the addition of increasing concentrations of Cys (0–3 μM) at pH 7.4 (phosphate buffer, 50 mM). Reaction time, 60 min. The inset shows a linear relationship between fluorescence intensity and Cys concentration with a correlation coefficient of 0.995.

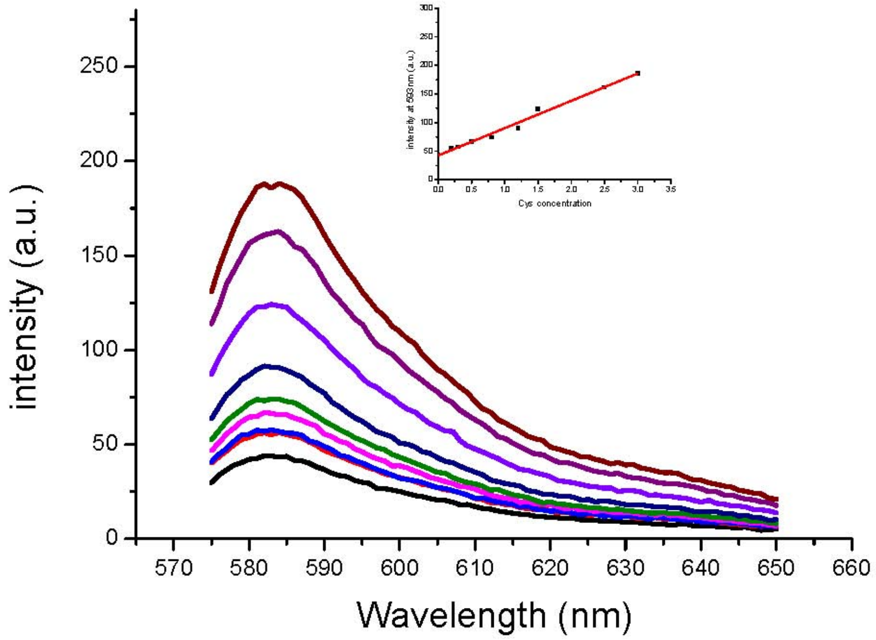

Figure S6. HRMS of the reaction mixture of **4** (20 μM) and Cys (20 μM) in 1:1 MeOH:H_2O showing formation of **3a**. Reaction time: 3 hours.

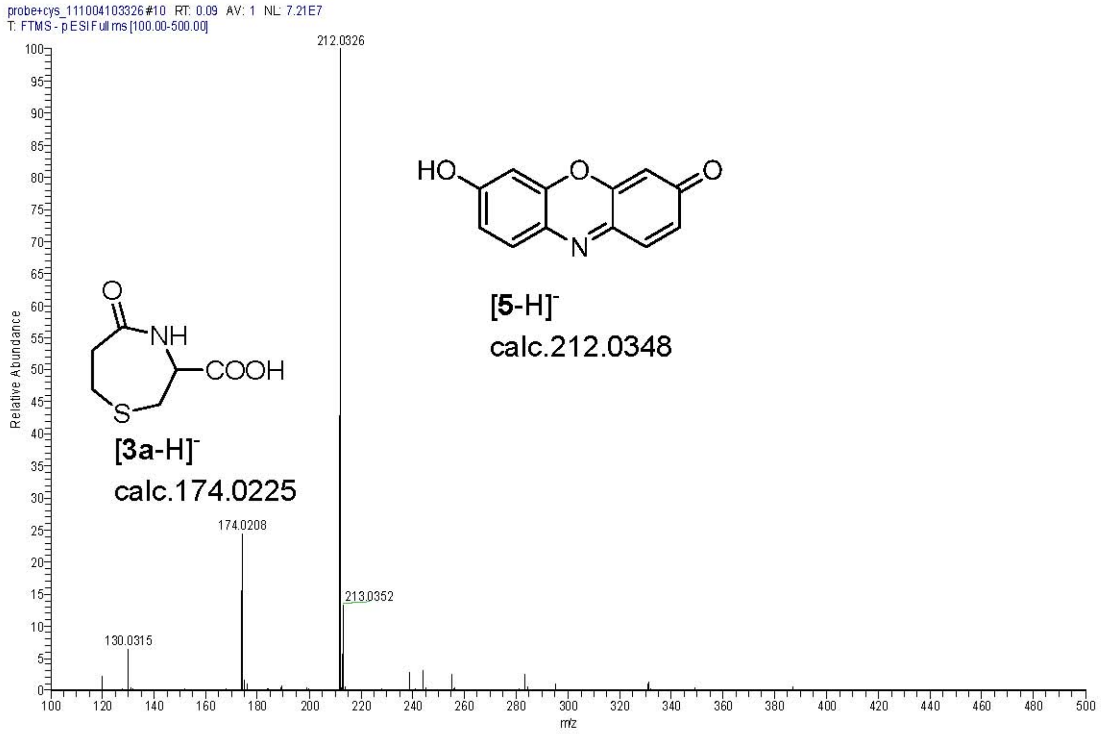

Figure S7. HRMS of the reaction mixture of **4** (20 μM) and Hcy (20 μM) in 1:1 MeOH:H_2O showing formation of **3b**. Reaction time: 3 hours.

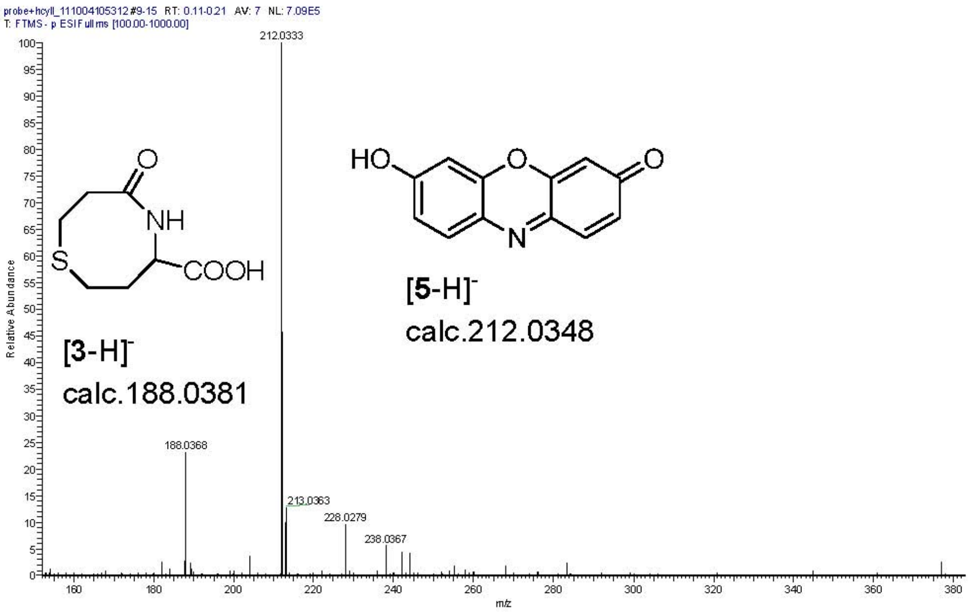

Figure S8. Response of **4** towards thiols upon inclusion of various surfactants. Time-dependent fluorescence changes ($\lambda_{ex}/\lambda_{em}$ = 565/590 nm) of **4** (2.5 μM) towards various thiols (2 equiv) in phosphate buffered media (50 mM, pH = 7.4) including surfactants (**a**) SDS (10 mM); (**b**) Triton X-100 (0.3 mM); (**c**) BC (0.05 mM; (**d**) CTAB (2 mM).

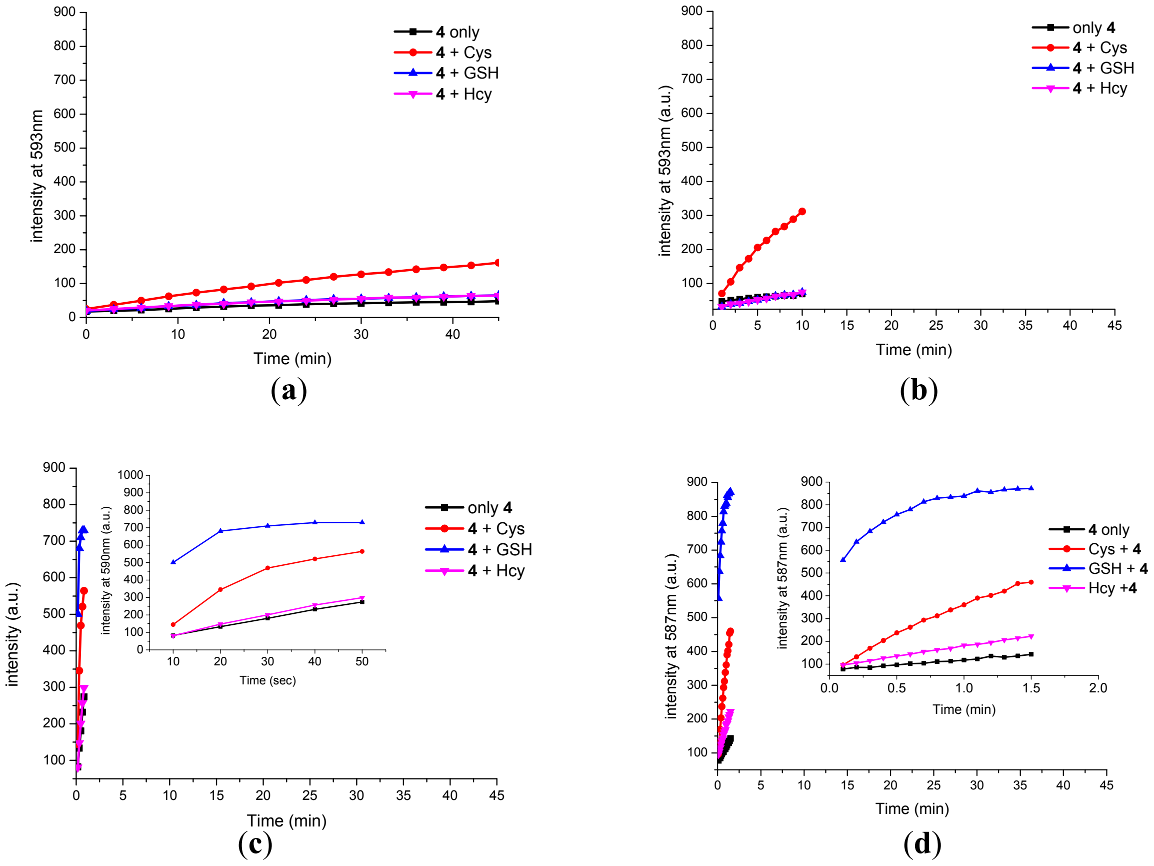

Figure S9 Surfactant-mediated response of the **4**-CTAB system towards GSH in the presence of Cys. Fluorescence spectra (λ_{ex} = 565 nm) of **4** (1.5 μM) upon the addition of a mixture of Cys (2 μM) and increasing concentrations of GSH (0–2 μM) at pH 7.4 (phosphate buffer, 50 mM). Reaction time, 2 min. The inset shows a linear relationship between fluorescence intensity and GSH concentration with a correlation coefficient of 0.990 and also between fluorescence intensity and a mixture GSH and Cys with a correlation coefficient of 0.9894.

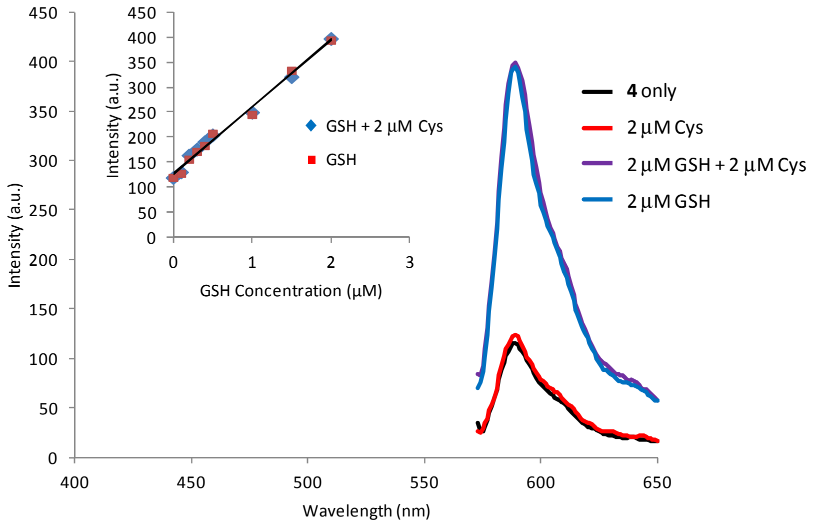

Figure S10. HRMS of the reaction mixture of **4** (20 μM) and GSH (20 μM) in 1:1 MeOH:H_2O showing the formation of **7**. Reaction time: 3 hours.

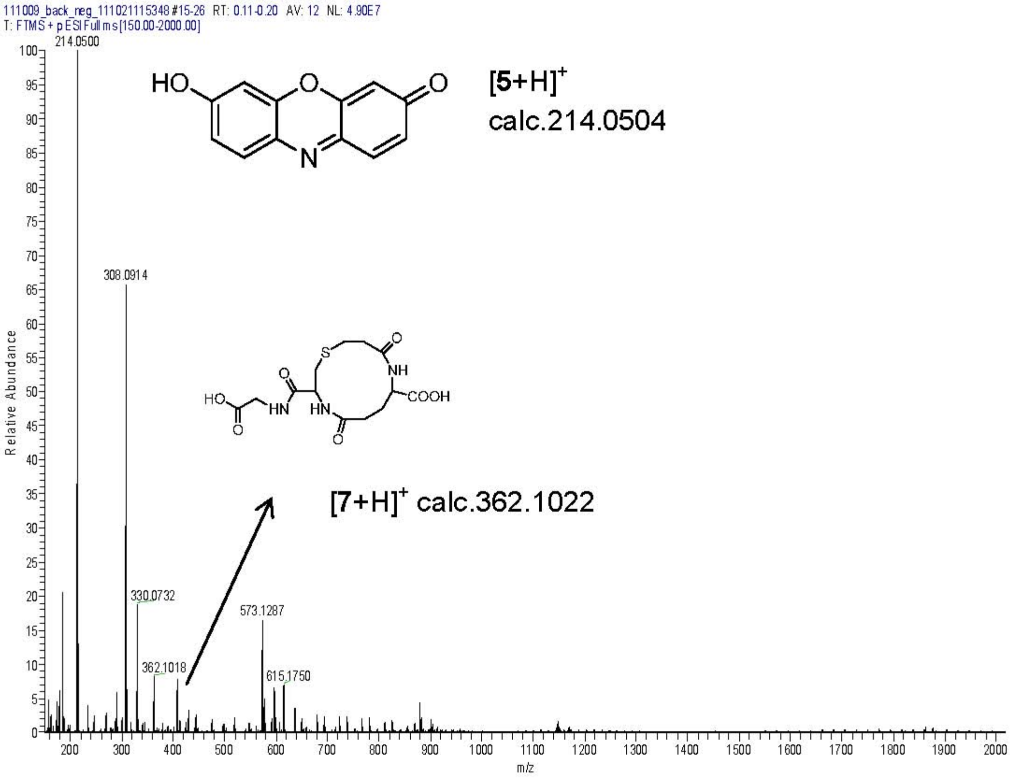

Figure S11. HRMS of the reaction mixture of **4** (20 µM) and GSH (20 µM) in 2.0 mM CTAB medium buffered at pH 7.4 (Hepes buffer, 20 mM) showing the formation of **7**. Reaction time: 5 min.

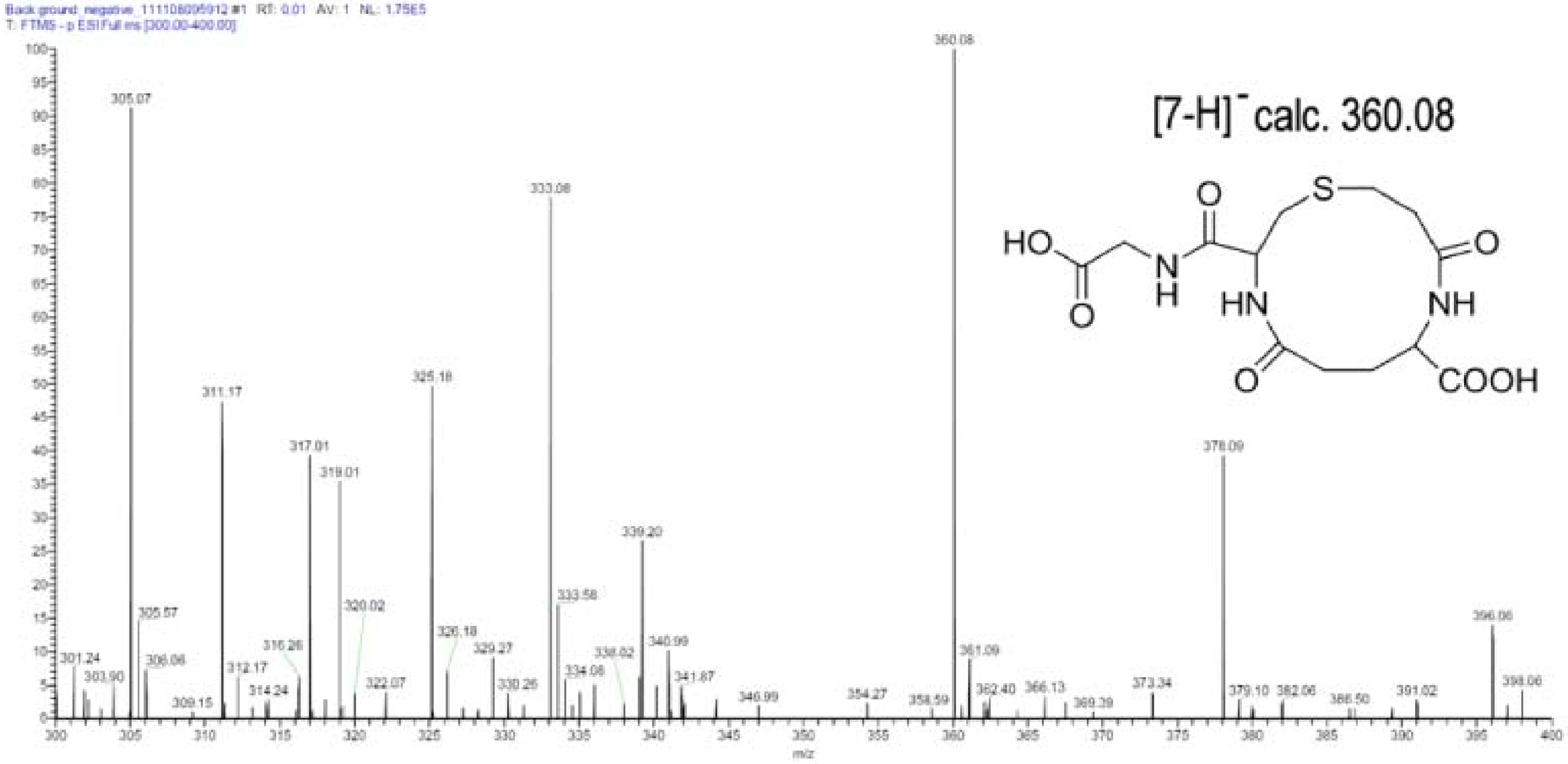

Figure S12. Spectral behavior of the **4**-CTAB system under different pH conditions (50 mM phosphate buffer). (**a**) Time-dependent absorbance changes of a solution of **4** (2.5 μM) at 580 nm in CTAB media (2 mM) buffered at pH 5.5 to 8; (**b**) Absorbance spectra of **4** in pH 6, 7, and 8 phosphate buffer after 1 min; (**c**) Absorbance spectra of **4** in pH 6, 7 and 8 phosphate buffer after 10 min.

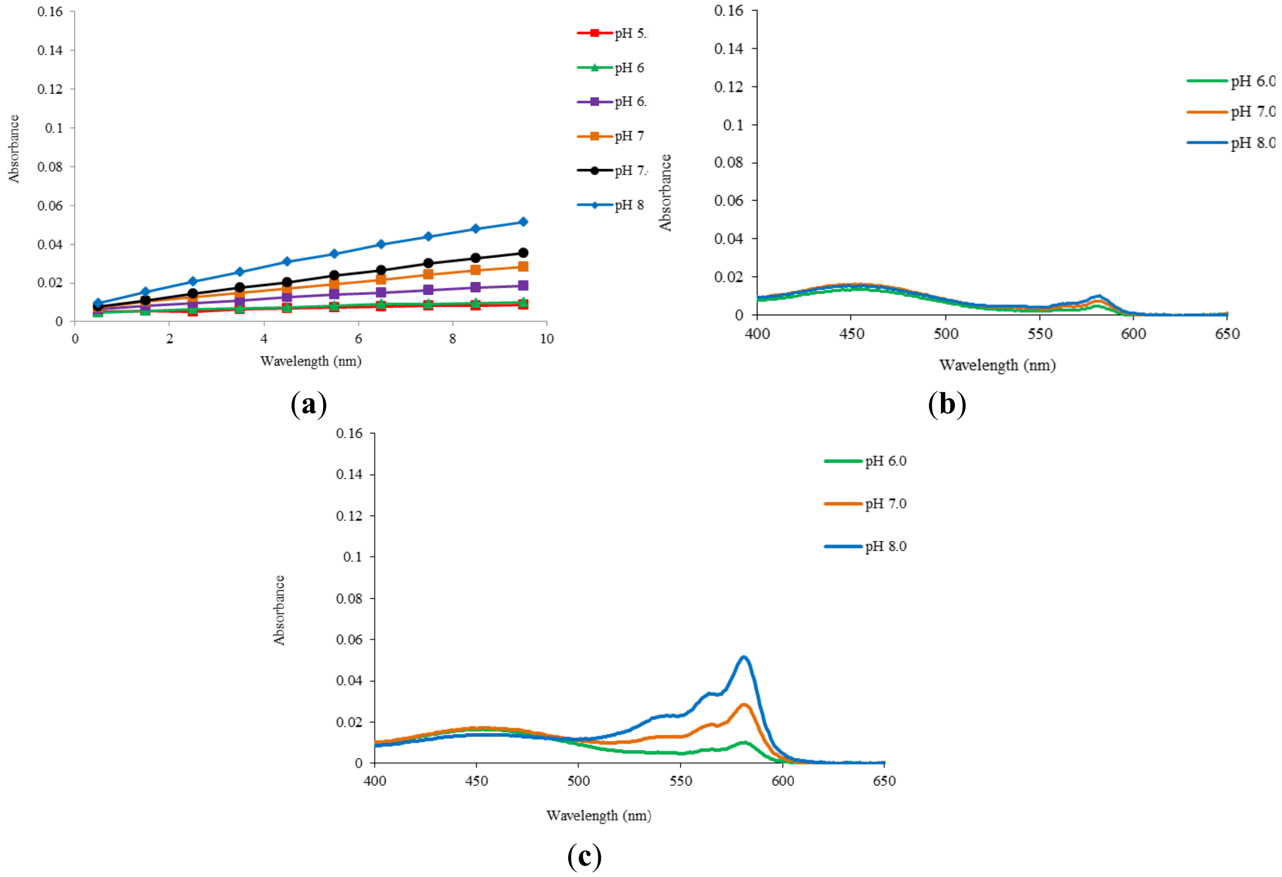

Figure S13. Fluorescence spectra (λex = 565 nm) of **4** (1.5 μM) upon addition of GSH (0–2.0 μM) and Cys (2 μM) to 10% deproteinized plasma diluted with 2.0 mM CTAB media buffered at pH 6.0 (phosphate buffer, 50 mM). The emission spectra were collected 8 min after mixing.

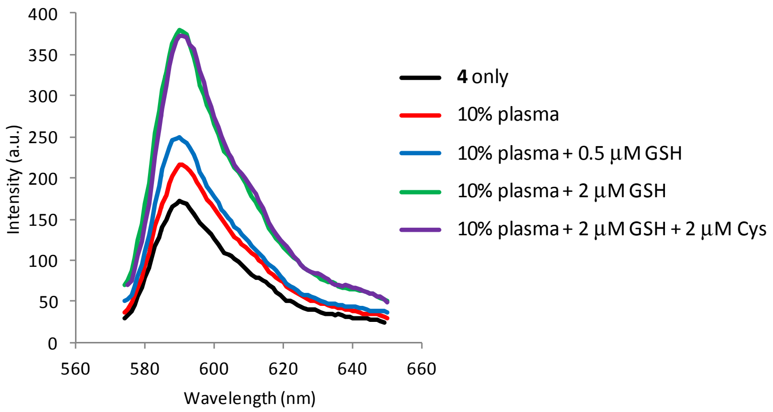

© 2012 by the authors; licensee MDPI, Basel, Switzerland. This article is an open access article distributed under the terms and conditions of the Creative Commons Attribution license (http://creativecommons.org/licenses/by/3.0/).
